# Prognostic Impact of ABO/Rh Blood Group–Systemic Inflammation Indices Interactions in Small-Cell Lung Cancer

**DOI:** 10.3390/biomedicines14051051

**Published:** 2026-05-06

**Authors:** Anıl Yıldız, Burak Alper Zengin, Maral Martin Mildanoglu, Bedirhan Ulufer, Ahmet Bilici, Naziye Ak

**Affiliations:** 1 Department of Medical Oncology, Basaksehir Cam ve Sakura City Hospital, 34480 İstanbul, Türkiye; anilyildiz@live.com; 2 Department of Medical Oncology, Institute of Oncology, Istanbul University, 34093 İstanbul, Türkiye; 3 Department of Medical Oncology, Faculty of Medicine, Medipol University, 34083 İstanbul, Türkiye

**Keywords:** small-cell lung cancer, ABO/Rh blood group, systemic inflammation, prognosis, treatment response

## Abstract

**Background/Objectives**: ABO/Rh blood groups and systemic inflammation are each linked to cancer biology and prognosis, yet their combined and interactive prognostic value has not been clarified in small-cell lung cancer (SCLC). We investigated the distribution of ABO/Rh blood groups in SCLC, their association with baseline complete blood count (CBC)–derived inflammatory indices, and the prognostic significance of blood group–inflammation interactions for treatment response and survival outcomes. **Methods**: This retrospective study included 158 patients with SCLC with available ABO/Rh typing and pretreatment CBC data. Neutrophil-to-lymphocyte ratio (NLR), platelet-to-lymphocyte ratio (PLR), systemic immune–inflammation index (SII), and systemic inflammation response index (SIRI) were calculated using standard formulas. Treatment response was assessed according to RECIST v1.1 and categorized as response (CR/PR) versus non-response (SD/PD). Progression-free survival (PFS) and overall survival (OS) were analyzed using Cox regression models incorporating interaction terms. **Results**: Patients with blood group O exhibited consistently lower baseline inflammatory indices compared with non-O blood groups. Non-O blood group status and higher SIRI independently predicted chemoradiotherapy non-response. Among the evaluated indices, SIRI demonstrated superior diagnostic performance compared with SII, NLR, and PLR. Similarly, non-O blood group status and higher SIRI were independently associated with increased risks of progression and mortality. In joint analyses, compared with blood group O and low SIRI (reference), the highest risk of progression was observed in patients with blood group AB × high SIRI (HR 14.67), followed by blood groups A × high SIRI (HR 10.38) and B × high SIRI (HR 7.95). A similar pattern was observed for mortality, with the highest risk in patients with blood group AB × high SIRI (HR 22.76), followed by blood groups A × high SIRI (HR 13.93) and B × high SIRI (HR 12.41). **Conclusions**: ABO blood group is associated with distinct inflammatory profiles in SCLC. Elevated SIRI independently predicts treatment non-response and adverse survival, and its prognostic effect varies by blood group, underscoring the role of host biology in modulating systemic inflammation.

## 1. Introduction

Small-cell lung cancer (SCLC) is a high-grade neuroendocrine malignancy with an aggressive clinical course, rapid growth kinetics, and a strong link to tobacco exposure [1]. Although many patients achieve an initial response to first-line therapy, early relapse is common and long-term survival remains limited [2]. In practice, prognosis is still largely anchored to stage (limited vs. extensive), performance status, and a small set of routine clinical variables—yet outcomes vary substantially even within similar clinical strata. This variability sustains interest in inexpensive, readily available biomarkers that can refine risk stratification at diagnosis and during systemic therapy [3].

Cancer-associated inflammation is closely intertwined with tumor progression, immune escape, and resistance to treatment, and peripheral blood counts provide a practical window into this biology. Ratios and composite indices derived from routine complete blood counts—most commonly the neutrophil-to-lymphocyte ratio (NLR), platelet-to-lymphocyte ratio (PLR), and systemic immune–inflammation index (SII)—have therefore been studied as prognostic tools in SCLC. A large systematic review and meta-analysis in SCLC reported that higher NLR is consistently associated with inferior overall survival, supporting the clinical relevance of systemic inflammatory state in this disease [4]. In parallel, a dedicated meta-analysis focused on SII concluded that elevated SII is associated with worse survival in SCLC, reinforcing the value of composite immune–inflammation measures [5]. More recent work in the immunotherapy era also suggests that SII retains prognostic utility among SCLC patients treated with immune checkpoint inhibitors [6].

ABO and Rh(D) blood groups represent stable, inherited host characteristics that are universally available in clinical records and vary across populations. ABO-related glycosylation patterns are expressed in multiple tissues and have been linked to cancer susceptibility and outcomes through pathways that overlap with inflammation, immune modulation, and tumor dissemination [7]. A recent epigenome-wide analysis in blood donors reported differential plasma levels by ABO [including higher soluble E-selectin and lower Von Willebrand Factor (VWF) in type O vs. non-O donors], reinforcing that ABO status can track measurable differences in inflammation-adjacent vascular biology [8]. VWF itself is increasingly recognized as a mediator at the inflammation–thrombosis interface, released upon endothelial activation and contributing to thrombo-inflammatory states [9,10]. Beyond vascular markers, ABO differences have also been explored in cytokine profiles in severe systemic inflammation, suggesting that blood group may correlate with distinct immune–inflammatory response patterns under stress states [11,12].

Despite these converging lines of evidence, previous studies have generally evaluated blood groups or inflammatory indices separately or have investigated their combined significance in tumor types other than SCLC [13,14]. However, in the SCLC population, the prognostic role of the interaction between ABO/Rh blood groups and systemic inflammation has not yet been investigated. We postulated that the prognostic significance of complete blood count (CBC)-derived inflammatory indices in patients with SCLC may be influenced by the potential association between ABO/Rh blood groups and distinct systemic inflammatory profiles. Therefore, this study aimed to assess the potential interaction between ABO/Rh blood groups and CBC–derived inflammatory indices in patients with SCLC and to examine its impact on prognosis.

## 2. Materials and Methods

This retrospective multicenter study was carried out in adult patients with SCLC who were followed up at the Istanbul University Oncology Institute and the Medipol University Oncology Department from January 2013 to December 2023. The study was conducted in accordance with the Declaration of Helsinki and was approved by the Ethics Committee of Istanbul University Faculty of Medicine (Date: 10 September 2024, Decision No: 2888472). Owing to the retrospective design, the Ethics Committee waived the requirement for obtaining informed consent.

### 2.1. Study Population

Over the study period, 248 patients with a confirmed diagnosis of SCLC were retrospectively screened. Patients were eligible if they were aged ≥18 years at the time of diagnosis, had a histopathological or cytological diagnosis of small cell lung cancer, had documented ABO and Rh blood group status in their medical records, and had available baseline demographic and clinical data. Additionally, patients were required to have accessible follow-up data sufficient to determine survival outcomes and to have undergone at least one clinical evaluation following diagnosis. Tumors were staged according to the American Joint Committee on Cancer (AJCC) TNM classification, 8th edition [15]. Because the study period extended from 2013 to 2023, some cases had originally been documented according to earlier staging frameworks in the medical records; however, for the purpose of the present analysis, all patients were reviewed and re-staged according to the AJCC 8th edition to ensure uniform stage classification across the cohort. All patients received either a carboplatin-based doublet (carboplatin + etoposide) or a cisplatin-based doublet (cisplatin + etoposide) as part of their treatment protocol.

Patients were excluded if they were younger than 18 years; lacked histopathological or cytological confirmation of small cell lung cancer; had a non-SCLC histology; had concurrent active malignancy; had active or chronic infection; had paraneoplastic syndrome; had severe hematologic abnormalities; had incomplete demographic or clinical data; were lost to follow-up immediately after diagnosis; had a major acute inflammatory event (e.g., major surgery, major trauma, or acute myocardial infarction); or had no available survival follow-up data.

### 2.2. Data Collection

All data were collected from the electronic hospital information system and patient medical records. The collected variables included baseline demographic and clinical characteristics such as age, sex, smoking status, comorbidities, ABO blood group, primary tumor site, stage of disease, chemotherapy regimen, sites of metastasis, presence of paraneoplastic syndromes, Ki-67 proliferation index, pretreatment complete blood count parameters, Eastern Cooperative Oncology Group (ECOG) performance status, treatment-related adverse events/toxicities, treatment response, and disease progression or death during follow-up.

Chemoradiotherapy referred to definitive combined-modality treatment administered during initial management in patients with limited-stage disease.

ABO and Rh(D) blood group data were retrieved from institutional laboratory/transfusion service records and were determined by routine standard serologic testing as part of clinical care.

Baseline hematologic and inflammatory parameters were obtained from pretreatment CBC measurements, defined as those collected within ±7 days of pathological diagnosis and before systemic therapy; when multiple values were available, the one nearest to the diagnosis date was chosen. The following indices were calculated from the same baseline sample using standard formulas: NLR = neutrophils/lymphocytes, PLR = platelets/lymphocytes, SII = (platelets × neutrophils)/lymphocytes, and systemic inflammation response index (SIRI) = (neutrophils × monocytes)/lymphocytes.

### 2.3. Outcomes

Tumor response was assessed according to Response Evaluation Criteria in Solid Tumors (RECIST) version 1.1. Computed tomography (CT) imaging was performed at baseline and every 12 weeks. Tumor response at 12 weeks were recorded and categorized as complete response (CR), partial response (PR), stable disease (SD), or progressive disease (PD).

Progression-free survival (PFS) was defined as the time from initiation of first-line systemic therapy to radiologically confirmed progression or death from any cause, whichever occurred first. Patients without progression or death at data cutoff were censored at the date of the last adequate tumor assessment. Overall survival (OS) was defined as the time from initiation of first-line systemic therapy to death from any cause; patients alive at data cutoff were censored at the date of last follow-up/contact.

### 2.4. Statistical Analysis

All analyses were conducted using IBM SPSS Statistics for Windows, version 25.0 (IBM Corp., Armonk, NY, USA). The normality of distribution for continuous variables was assessed using the Kolmogorov–Smirnov test. Normally distributed variables were presented as mean ± standard deviation (SD), and non-normally distributed variables as median and interquartile range (IQR: 25th–75th percentiles). Comparisons between two independent groups were performed using Student’s *t*-test for normally distributed variables and the Mann–Whitney U test for non-normally distributed variables. For comparisons involving more than two groups, one-way analysis of variance (ANOVA) or the Kruskal–Wallis test was used, depending on the distribution. Categorical variables were expressed as counts and percentages and compared using the Chi-square or Fisher’s exact test, as appropriate. Time-to-event outcomes, including progression-free survival (PFS) and overall survival (OS), were estimated using the Kaplan–Meier method and compared between groups using the log-rank test. Prognostic factors for PFS and OS were evaluated using univariable and multivariable Cox proportional hazards regression analyses. Variables found to be significant in univariable regression analysis were included in multivariate models. Hazard ratios (HRs) and 95% confidence intervals (CIs) were reported. Receiver operating characteristic (ROC) curve analyses were then used to assess the discriminative performance of inflammatory indices for predicting outcomes. Optimal cutoff values were derived using the Youden index, and differences between AUCs of independent predictors were compared with the DeLong test. A two-tailed *p*-value < 0.05 was considered statistically significant for all analyses.

## 3. Results

A total of 158 SCLC patients were included, with a mean age of 61.9 ± 9.3 years, and the majority were male (73.4%). Comorbidities were present in 57.0% of patients, with hypertension being the most common (41.1%), followed by diabetes mellitus (20.9%) and coronary artery disease (18.4%). The median Charlson Comorbidity Index (CCI) score was 8.0 (IQR 6.0–9.0). Most patients were Rh-positive (88.6%), and blood group A was the most frequent (40.5%). At diagnosis, the majority of patients had advanced-stage disease, with 58.9% presenting with stage IV and 35.4% with stage III disease. Central tumor localization was observed in 36.1% of cases. Metastatic disease was present in 73.4% of patients, most commonly involving bone (42.4%) and liver (28.5%), followed by brain (20.3%) and adrenal glands (13.3%). The majority had good performance status (ECOG 0–1: 96.9%). Cisplatin-based doublet chemotherapy was administered in 60.8% of patients, while 39.2% received carboplatin-based regimens. At 6 months, 73.4% of patients achieved a treatment response. Grade 3–4 toxicity occurred in 29.1% of patients. During follow-up, 45.6% experienced disease progression and 53.8% died. The median PFS was 52 months (95% CI = 45–60), and the median follow-up duration was 53 months (95% CI = 45–60) (Supplementary Table S1).

### 3.1. Findings Related to ABO Blood Group

When patients were stratified according to ABO blood group (O vs. non-O), male gender was less frequent in the O group (61.2% vs. 78.9%; *p* = 0.020). The O group had lower CCI scores (6.0 vs. 8.0; *p* = 0.001) and a lower Rh (+) positivity rate (77.6% vs. 93.6%; *p* = 0.003). Inflammatory markers were significantly lower in the O group, including Neutrophils (5.2 vs. 6.2 × 10^9^/L; *p* = 0.034), monocytes (0.6 vs. 0.7 × 10^9^/L; *p* = 0.012), SII (792.0 vs. 1133.7; *p* = 0.008), SIRI (1.7 vs. 2.4; *p* = 0.003), NLR (2.5 vs. 3.4; *p* = 0.012), and CRP (6.4 vs. 13.0 mg/L; *p* = 0.001) (Table 1).

**Figure 1:**
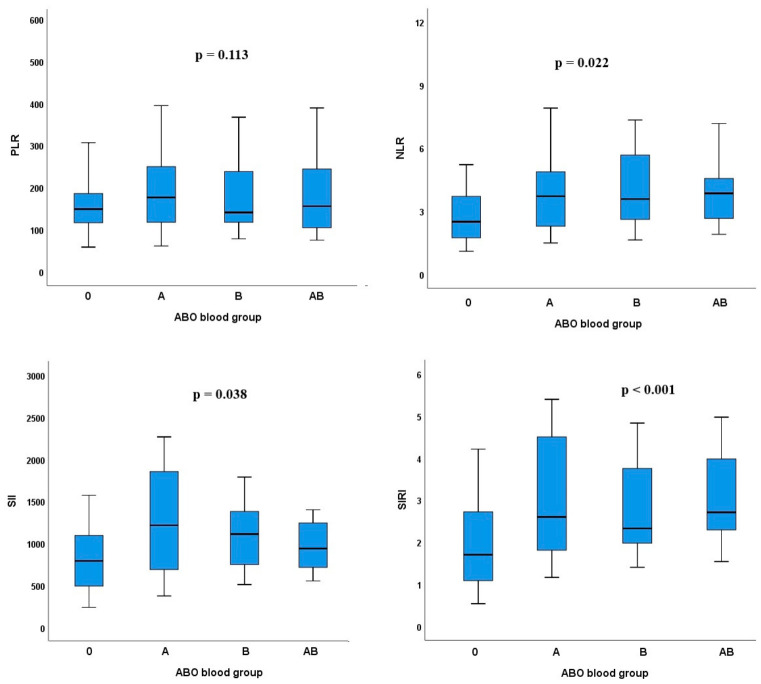
Distribution of inflammatory indices according to ABO blood groups in SCLC patients.

Median NLR (O: 2.5 vs. A: 3.7 vs. B: 3.4 vs. AB: 3.9; *p* = 0.022), SII (O: 792 vs. A: 1216 vs. B: 1027 vs. AB: 939; *p* = 0.038), and SIRI (O: 1.7 vs. A: 2.6 vs. B: 2.3 vs. AB: 2.7; *p* < 0.001) differed significantly across ABO blood groups, with non-O groups generally demonstrating higher values compared to blood group O. In contrast, median PLR values were comparable among groups (O: 148.6 vs. A: 176.2 vs. B: 140.6 vs. AB: 159.1; *p* = 0.113) (Figure 1).

Central tumor localization was less frequent in the O blood group (24.5% vs. 41.3%; *p* = 0.042). Advanced-stage disease (stage IV) was less common among patients with blood group O (38.8% vs. 67.9%; *p* = 0.001), and liver metastasis was also less frequent (14.3% vs. 34.9%; *p* = 0.008). ECOG performance status differed significantly (*p* = 0.001), with ECOG 0 more frequent in the O group. Grade 3–4 toxicity was less common in the O group (16.4% vs. 33.9%; *p* = 0.046). Treatment response at 6 months was significantly better in patients with blood group O, with a lower proportion of non-responders (8.2% vs. 34.9%; *p* < 0.001). Disease progression (26.5% vs. 54.1%; *p* < 0.001) and mortality (28.6% vs. 65.1%; *p* < 0.001) were also significantly lower in the O group (Table 2).

### 3.2. Diagnostic Performance of Inflammatory Indices for Predicting Outcomes

Median SII, SIRI, and NLR levels were higher in non-responders (SII: 1372.1 vs. 879.8; *p* < 0.001; SIRI: 4.2 vs. 1.9; *p* < 0.001; NLR: 4.5 vs. 2.8; *p* < 0.001). In contrast, median PLR values were comparable among groups (Supplementary Table S2). Patients with disease progression had higher median inflammatory indices compared to those without progression, including SII (1181.7 vs. 726.8; *p* < 0.001), SIRI (3.5 vs. 1.7; *p* < 0.001), NLR (3.8 vs. 2.5; *p* < 0.001), and PLR (182.0 vs. 148.7; *p* = 0.012) (Supplementary Table S3). Similarly, non-survivors demonstrated elevated inflammatory markers relative to survivors, with higher median SII (1243.1 vs. 637.5; *p* < 0.001), SIRI (3.5 vs. 1.5; *p* < 0.001), NLR (4.0 vs. 2.3; *p* < 0.001), and PLR (182.3 vs. 138.1; *p* = 0.008) levels (Supplementary Table S4).

ROC analysis demonstrated that SIRI consistently showed the highest diagnostic performance across all outcomes. For chemotherapy response, SIRI yielded an AUC of 0.73 with 72.0% sensitivity and 81.9% specificity. In predicting disease progression, SIRI achieved an AUC of 0.74 with 79.2% sensitivity and 70.3% specificity. For mortality, SIRI demonstrated the best discriminative ability with an AUC of 0.80, 77.1% sensitivity, and 84.9% specificity (Table 3).

To avoid potential multicollinearity among inflammatory indices in the multivariable regression models for outcomes, SIRI was selected for inclusion given its superior diagnostic performance across all endpoints (Table 3). Additionally, considering the differences observed in inflammatory indices according to ABO blood groups, the interaction between ABO blood group and SIRI was also used in the multivariable regression model for all endpoints.

### 3.3. Independent Factors Associated with Response to Chemotherapy at 6 Months

Male gender was more frequent among responders compared to non-responders (69.0% vs. 58.7%; *p* = 0.035). Blood group O was more common among responders (38.8% vs. 9.5%), whereas blood groups A (47.6% vs. 37.9%), B (33.3% vs. 17.2%), and AB (9.5% vs. 6.0%) were more frequent in non-responders (*p* = 0.002). Inflammatory indices including SII, SIRI, and NLR were markedly elevated in non-responders (SII: 1372.1 vs. 879.8; *p* < 0.001; SIRI: 4.2 vs. 1.9; *p* < 0.001; NLR: 4.5 vs. 2.8; *p* < 0.001), along with higher CRP levels (32.5 vs. 8.0 mg/L; *p* < 0.001). Stage IV disease was more common in non-responders (85.7% vs. 49.1%; *p* < 0.001), liver metastasis was more frequent (50.0% vs. 20.7%; *p* < 0.001), and ECOG performance status differed significantly between groups (*p* < 0.001). Chemoradiotherapy prior to metastasis was more common among responders (40.5% vs. 16.7%; *p* = 0.005). Progression and mortality rates were higher in non-responders (progression: 73.8% vs. 35.3%; *p* < 0.001; mortality: 90.5% vs. 40.5%; *p* < 0.001), and both PFS (28.0 vs. 60.0 months; *p* < 0.001) and OS (28.0 vs. 60.0 months; *p* = 0.002) were shorter in this group. Other demographic and clinical variables did not differ significantly between responders and non-responders (*p* > 0.05) (Supplementary Table S2).

Potential risk factors associated with non-response to chemotherapy are presented in Table 4. In the multivariable logistic regression analysis, ABO blood group remained independently associated with non-response. Compared with blood group O, patients with blood groups A (OR 3.68, 95% CI 1.07–12.64; *p* = 0.019), B (OR 8.72, 95% CI 2.30–32.93; *p* = 0.002), and AB (OR 4.94, 95% CI 1.03–29.31; *p* = 0.028) exhibited significantly higher odds of non-response. Higher SIRI levels (OR 1.44, 95% CI 1.09–1.90; *p* = 0.010) and CRP levels (OR 1.03, 95% CI 1.01–1.04; *p* = 0.011) were also independently associated with increased likelihood of non-response. Furthermore, stage IV disease (OR 4.43, 95% CI 1.39–14.05; *p* = 0.009) and ECOG 1–2 status (OR 23.44, 95% CI 8.64–63.58; *p* < 0.001) remained significantly associated with non-response in the multivariable model. The interaction terms for A × SIRI (OR 1.45, 95% CI 1.03–2.26; *p* = 0.016), B × SIRI (OR 1.85, 95% CI 1.19–2.88; *p* = 0.008), and AB × SIRI (OR 1.71, 95% CI 1.17–2.51; *p* = 0.011) indicate that the association between SIRI and non-response varied according to ABO blood group.

**Figure 2:**
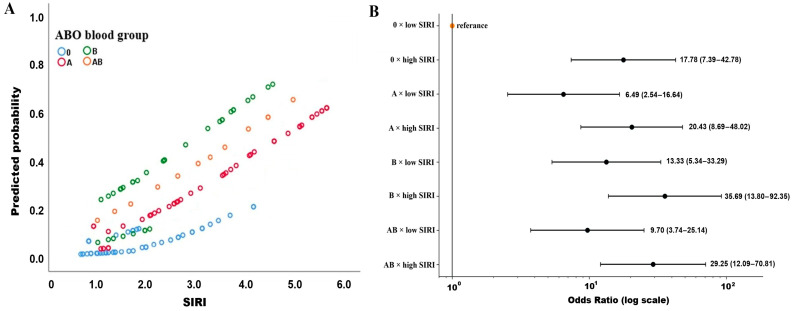
Interaction between ABO blood group and SIRI in relation to non-response to chemotherapy. (**A**) Predicted probability of non-response according to continuous SIRI levels across ABO blood groups, derived from the multivariable logistic regression model. (**B**) Joint association of ABO blood group and SIRI (dichotomized using the ROC-derived cut-off > 2.1) with non-response to chemotherapy. Odds ratios (ORs) and 95% confidence intervals (CIs) are shown, with blood group O and low SIRI as the reference category.

In line with the significant interaction observed in the regression analysis, the association between SIRI and non-response differed across ABO blood groups (Figure 2). As shown in Figure 2A, the predicted probability of non-response increased with rising SIRI levels in all groups; however, the slope of this increase was more pronounced in non-O blood groups, particularly in blood group B. Figure 2B further demonstrated the joint effect of ABO blood group and dichotomized SIRI (ROC-derived cut-off, see Table 3), revealing a stepwise increase in non-response risk among patients with high SIRI levels across non-O groups. The highest risk was observed in patients with blood group B × high SIRI (OR 35.69, 95% CI 13.80–92.35), followed by blood groups AB and A with high SIRI. These findings visually corroborate the statistically significant ABO × SIRI interaction identified in the multivariable model.

### 3.4. Independent Factors Associated with Disease Progression

In crude regression analysis, higher CCI score, non-O blood groups, Rh positivity, elevated inflammatory markers (neutrophils, monocytes, SII, SIRI, NLR, PLR, and CRP), stage IV disease, liver and bone metastases, worse ECOG performance status, and non-response to chemotherapy were associated with disease progression. Peripheral tumor localization was associated with lower progression risk (Supplementary Table S3).

Selected potential risk factors associated with disease progression are presented in Table 5. In the multivariable Cox regression analysis, several factors remained independently associated with disease progression. Compared with blood group O, blood group A was associated with a 2.55-fold higher risk of progression (HR = 2.55, 95% CI = 1.31–4.68; *p* = 0.005), blood group B with a 2.48-fold higher risk (HR = 2.48, 95% CI = 1.15–4.29; *p* = 0.015), and blood group AB with a 6.91-fold higher risk (HR = 6.91, 95% CI = 2.70–17.68; *p* = 0.003). Each one-unit increase in SIRI was associated with a 1.24-fold higher risk of progression (HR = 1.24, 95% CI = 1.11–1.39; *p* < 0.001), each one-unit increase in CCI with a 1.38-fold higher risk (HR = 1.38, 95% CI = 1.18–1.60; *p* < 0.001), and each one-unit increase in CRP with a 1.06-fold higher risk (HR = 1.06, 95% CI = 1.02–1.11; *p* = 0.005). Stage IV disease was associated with a 3.61-fold higher risk of progression (HR = 3.61, 95% CI = 2.08–6.26; *p* < 0.001), ECOG 1–2 with a 2.45-fold higher risk (HR = 2.45, 95% CI = 1.56–3.84; *p* = 0.001), and non-response to chemotherapy with a 2.04-fold higher risk (HR = 2.04, 95% CI = 1.17–3.55; *p* = 0.012). In contrast, peripheral tumor localization was associated with a lower risk of progression than central localization (HR = 0.48, 95% CI = 0.31–0.76; *p* = 0.008). Importantly, the significant ABO blood group × SIRI interaction indicated that the adverse effect of increasing SIRI on progression risk was not constant across blood groups but was stronger in non-O patients; relative to blood group O, this effect was 1.33-fold stronger in blood group A, 1.22-fold stronger in blood group B, and 1.55-fold stronger in blood group AB (Table 5).

**Figure 3:**
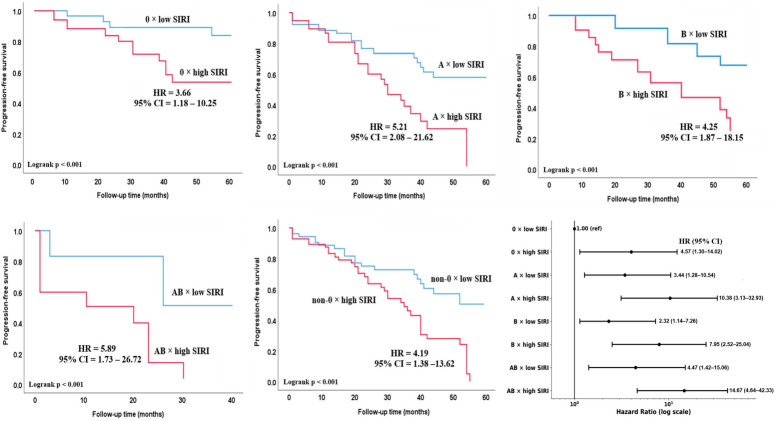
Interaction between ABO blood group and SIRI in relation to PFS in patients with SCLC. Kaplan–Meier curves illustrate PFS stratified by ABO blood group and SIRI levels. SIRI was dichotomized according to the ROC-derived cut-off value (low vs. high). Within each ABO subgroup, patients with high SIRI exhibited a higher risk of progression compared with those with low SIRI. The joint association of ABO blood group and SIRI is additionally presented in the forest plot, with blood group O and low SIRI serving as the reference category. Hazard ratios (HRs) and 95% confidence intervals (CIs) were derived from the adjusted Cox regression model.

Kaplan–Meier analysis demonstrated that the risk of disease progression was significantly higher in patients with high SIRI across all ABO blood groups (all log-rank *p* < 0.001; Figure 3). However, the magnitude of this association varied by blood type. High SIRI (≥2.3, see Table 3) was associated with increased progression risk in blood group O (HR 3.66), A (HR 5.21), B (HR 4.25), and AB (HR 5.89). In the Forest plot analysis, compared with blood group O × low SIRI (reference), the highest risk was observed in patients with blood group AB × high SIRI (HR 14.67), followed by blood groups A × high SIRI (HR 10.38) and B × high SIRI (HR 7.95).

### 3.5. Independent Factors Associated with Mortality

In crude regression analysis, higher CCI score, non-O blood groups, Rh positivity, elevated inflammatory markers (leukocytes, monocytes, SII, SIRI, NLR, PLR, and CRP), stage IV disease, liver, bone, and adrenal metastases, worse ECOG performance status, non-response to chemotherapy, and disease progression were associated with increased mortality. Peripheral tumor localization and prior chemoradiotherapy were associated with lower mortality risk (Supplementary Table S4).

Potential risk factors associated with mortality are presented in Table 6. In the multivariable Cox regression analysis, both ABO blood group and SIRI remained independently associated with mortality. Compared with blood group O, blood groups A (HR 1.98, 95% CI 1.04–3.78; *p* = 0.022), B (HR 2.04, 95% CI 1.04–4.01; *p* = 0.024), and AB (HR 3.48, 95% CI 1.29–9.72; *p* = 0.014) were associated with increased mortality risk. Higher SIRI levels were also independently associated with mortality (HR 1.14, 95% CI 1.03–1.25; *p* = 0.011). A significant interaction between ABO blood group and SIRI was observed in the adjusted model (A × SIRI: HR 1.23, 95% CI 1.02–1.48; *p* = 0.027; B × SIRI: HR 1.21, 95% CI 1.02–1.38; *p* = 0.032; AB × SIRI: HR 1.47, 95% CI 1.08–2.00; *p* = 0.016). Additionally, male gender, CCI, tumor location, TNM stage IV, ECOG 1-2 status, non-response to chemotherapy, and disease progression remained significantly associated with mortality in the multivariable model (Table 6).

**Figure 4:**
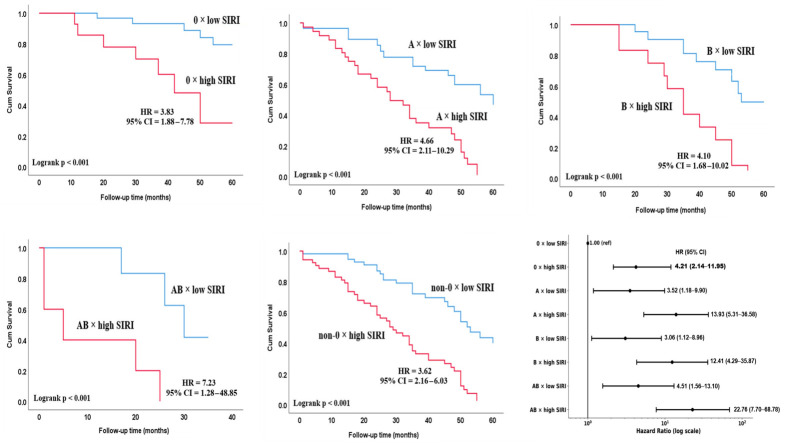
Interaction between ABO blood group and SIRI in relation to mortality in patients with SCLC. Kaplan–Meier curves illustrate PFS stratified by ABO blood group and SIRI levels. SIRI was dichotomized according to the ROC-derived cut-off value (low vs. high). Within each ABO subgroup, patients with high SIRI exhibited a higher risk of mortality compared with those with low SIRI. The joint association of ABO blood group and SIRI is additionally presented in the forest plot, with blood group O and low SIRI serving as the reference category. Hazard ratios (HRs) and 95% confidence intervals (CIs) were derived from the adjusted Cox regression model.

Kaplan–Meier analysis demonstrated that the risk of mortality was significantly higher in patients with high SIRI across all ABO blood groups (all log-rank *p* < 0.001; Figure 4). The magnitude of this association varied according to blood type. High SIRI (≥2.6, see Table 3) was associated with increased mortality risk in blood group O (HR 3.83), A (HR 4.66), B (HR 4.10), and most prominently in blood group AB (HR 7.23). In the Forest plot analysis, compared with patients with blood group O and low SIRI (reference), the highest mortality risk was observed in patients with blood group AB × high SIRI (HR 22.76), followed by blood groups A × high SIRI (HR 13.93) and B × high SIRI (HR 12.41).

## 4. Discussion

To the best of our knowledge, this study is the first to investigate the interaction between ABO blood group and systemic inflammation in patients with SCLC. Systemic inflammatory indices were significantly higher in non-O blood groups compared with blood group O. Patients with treatment non-response and poorer survival outcomes more frequently exhibited non-O blood groups and elevated inflammatory indices. Moreover, regression models incorporating the interaction between ABO blood group and systemic inflammatory indices enabled clearer identification of high-risk subgroups for treatment non-response and adverse survival outcomes. These findings suggest that ABO-related host factors may influence the systemic inflammatory milieu and thereby modulate treatment sensitivity and survival in SCLC.

A systematic review and meta-analysis of multiple studies reported a positive association between blood group A and overall cancer risk, while blood group O appeared to be negatively associated [16]. However, the existing literature lacks studies assessing the prognostic relevance of blood groups in SCLC cohorts. Non-O blood groups were more common in patients with treatment resistance and worse outcomes, indicating that ABO status may reflect host heterogeneity in SCLC. Similar patterns involving non-O blood groups have also been observed across other lung cancer subtypes [17,18,19]. Nevertheless, in metastatic NSCLC treated with pembrolizumab monotherapy, blood group O has been reported to associate with more favorable survival, whereas this association was not observed in chemo-immunotherapy or chemotherapy cohorts [18]. On the other hand, the effect of ABO blood groups on outcomes has been reported to exhibit variability across different cancer subtypes [20,21,22,23]. These observations underscore that ABO-related host factors may modulate coagulation, endothelial activation, and immune–inflammatory phenotypes, and that the net clinical impact of ABO may depend on tumor type and treatment context [24,25]. Therefore, the mechanisms and clinical utility of ABO-informed stratification warrant further investigation in SCLC.

To better contextualize ABO-related host heterogeneity in SCLC, we examined a panel of CBC-based systemic inflammatory indices. CBC-based systemic inflammatory indices were higher in patients with treatment resistance and worse survival outcomes. Furthermore, SIRI outperformed the other inflammatory indices in terms of prognostic value. A substantial body of evidence supports systemic inflammation indices as prognostic markers in SCLC, although comparative performance across indices has been inconsistent. A recent systematic review and meta-analysis in patients receiving first-line platinum-based chemotherapy found that elevated NLR was associated with worse overall survival and progression-free survival, whereas PLR showed weaker or subgroup-dependent associations [26]. A broader systematic review of inflammation scores in SCLC similarly supports prognostic utility—particularly for NLR—while emphasizing heterogeneity across studies and thresholds [4]. In stage III non- SCLC, SII has been reported to predict chemoradiation resistance and poor survival, and to outperform other inflammation-based factors in prognostic performance [27]. Complementing these data, a dedicated meta-analysis focusing on SII in SCLC concluded that higher SII is associated with poorer overall survival [5]. Within SCLC cohorts, comparative analyses that include multiple indices also suggest that incorporating monocyte-related information can add prognostic value; in extensive-stage SCLC, SIRI has emerged as an independent predictor for both progression-free and overall survival in multivariable models [28]. Similarly, in limited-stage SCLC specifically, pretreatment SIRI has been shown to stratify overall survival in patients receiving concurrent chemoradiotherapy [29]. In unresectable stage III non-SCLC treated with chemoradiotherapy, pretreatment SIRI independently predicted overall survival and was reported to be prognostically superior to NLR [30]. Recent histology-based analyses evaluating multiple CBC-derived inflammatory indices across lung cancer subtypes have demonstrated that inflammatory profiles evolve in parallel with disease progression. Notably, SIRI has been reported to be elevated in SCLC and identified as a predictor of disease severity [31,32]. Because SIRI has also shown adverse prognostic associations across diverse cancers in meta-analytic evidence, its use as a cross-tumor risk signal appears biologically coherent [33]. However, the diagnostic performance of systemic inflammatory indices may be influenced by interindividual differences in host biology.

**Figure 5:**
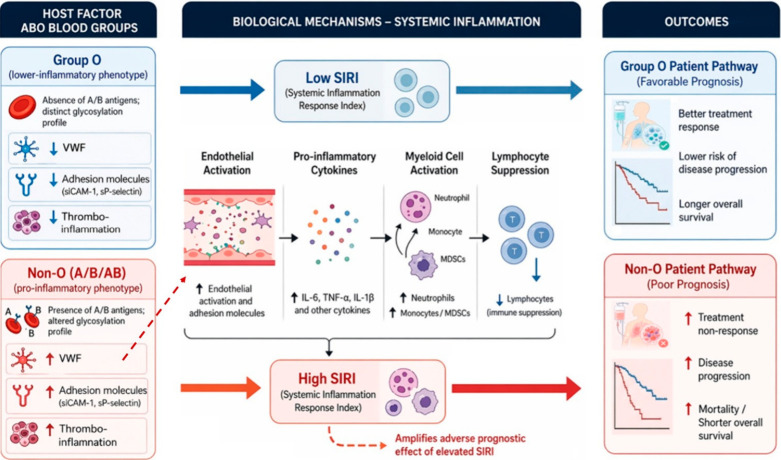
Conceptual framework of the ABO blood group–systemic inflammation interaction in SCLC. Patients with blood group O are illustrated as having a lower-inflammatory profile, including lower VWF, reduced endothelial adhesion signaling, less thrombo-inflammation, and lower SIRI, which are associated with a more favorable clinical course. In contrast, non-O blood groups (A/B/AB) are associated with a pro-inflammatory phenotype marked by enhanced adhesion molecule activity, cytokine release, myeloid cell activation, and relative lymphocyte suppression, resulting in higher SIRI. These inflammatory differences may contribute to divergent clinical trajectories, with group O linked to more favorable outcomes and non-O groups linked to treatment resistance, disease progression, and shorter survival. Upward arrows denote relative increase, whereas downward arrows denote relative decrease.

In this study, patients with blood group O exhibited lower levels of systemic inflammatory indices, particularly SIRI, than those with non-O ABO blood groups, suggesting that ABO status may represent a stable host determinant of baseline systemic inflammation. Several biologically plausible pathways support this association. ABO antigens are glycans, and ABO-encoded glycosyltransferases shape glycosylation patterns that can modulate inflammation, cell–cell adhesion, and immune recognition—mechanisms broadly linked to inflammatory responses and metastatic behavior [34]. In population-based human studies, ABO variation has also been associated with differences in circulating soluble adhesion molecules involved in inflammatory cell trafficking (including sICAM-1 and sP-selectin) [35]. In addition, an umbrella review synthesizing evidence across many clinical phenotypes highlights that ABO/Rh loci are connected to broad systemic biology and can be associated with variation in multiple plasma proteins, including proteins with pro- and anti-inflammatory functions [36]. A particularly well-established axis is VWF, for which group O generally exhibits lower circulating levels than non-O individuals; given VWF’s roles in platelet–leukocyte interactions and inflammatory signaling [37], and its emerging links to cancer progression and metastasis [38], higher VWF-related thrombo-inflammatory activity in non-O patients could plausibly contribute to elevated CBC-derived inflammatory indices. Given that SCLC is a high-grade neuroendocrine carcinoma with a recognized tendency toward coagulation abnormalities, and that prior studies have demonstrated increased procoagulant activity and inducible tissue factor expression in SCLC, this disease may be particularly sensitive to host-level ABO–VWF-related thrombo-inflammatory differences [39,40]. Consistent with this, non-O status has been associated with increased risk of cancer-associated venous thromboembolism, with evidence supporting an ABO–VWF contribution to thrombosis heterogeneity in cancer [41]. These pathways also provide a mechanistic bridge to SIRI, which reflects neutrophil/monocyte predominance relative to lymphocytes. Myeloid-lineage programs—including tumor-associated macrophages and myeloid-derived suppressor cell phenotypes—can inhibit effector T-cell function, reinforce checkpoint-inducing and immunosuppressive mediators, and remodel the tumor microenvironment, ultimately enabling immune evasion, limiting therapeutic responsiveness, and worsening prognosis [42]. The proposed interplay between ABO status, systemic inflammation, and clinical outcomes is illustrated in Figure 5.

From a clinical perspective, incorporating an ABO × SIRI interaction term suggests that the prognostic impact of elevated SIRI is not uniform across patients but varies according to ABO blood group. In other words, the adverse implication of systemic inflammation appears to differ by blood type, supporting an effect modification framework rather than two independent additive predictors. This distinction is important, as ABO–cancer associations may appear inconsistent across studies when inflammatory status is not accounted for; if the biological influence of ABO is expressed primarily under specific inflammatory conditions, models considering only main effects may underestimate or overlook this relationship. Evidence from other settings supports the value of such integrative thinking. In gastric cancer, combining ABO status with CBC-derived inflammatory markers (including NLR-based measures) improved mortality risk stratification, and group O showed longer survival in that cohort [14]. In a retrospective stereotactic body radiotherapy cohort of lung cancer, post-treatment circulating lymphocyte counts were independently associated with short-term efficacy, and ABO status further stratified outcomes: patients with blood type A had shorter progression-free and overall survival than non-A groups, while non-A patients with higher post-treatment lymphocyte counts experienced the most favorable survival [43]. On the other hand, although the highest joint risk estimates were observed in the AB × high SIRI subgroup, these subgroup-specific findings should be interpreted cautiously because the number of patients with blood group AB was limited in our cohort. Additionally, patients with blood group O also had lower CCI scores at baseline, suggesting a lower overall comorbidity burden. Therefore, the more favorable outcomes observed in this subgroup may reflect, at least in part, lower systemic frailty in addition to a less pronounced inflammatory phenotype. Although CCI was incorporated into multivariable models, residual confounding related to baseline health status cannot be completely excluded.

From a practical standpoint, these findings are appealing because ABO blood group and pretreatment SIRI are inexpensive, rapidly available, and routinely collected in standard oncologic workflows. If externally validated, the interaction model could support pretreatment risk stratification in which non-O patients with elevated SIRI are considered a higher-risk subgroup for chemoradiotherapy non-response and early progression, potentially motivating closer radiographic surveillance, earlier evaluation of alternative systemic strategies, and/or prioritization for clinical trials targeting high-risk disease biology. In addition, although ROC-derived SIRI thresholds differed modestly according to the endpoint analyzed, the lowest observed threshold (>2.1) may have pragmatic value as an early-risk indicator for closer clinical follow-up, since it identified patients at risk beginning from treatment non-response. Nevertheless, this interpretation remains exploratory, and future studies should determine whether a unified threshold can be prospectively validated for simplified clinical risk stratification.

Several limitations should be considered when interpreting these findings. First, as this is a retrospective analysis, causality cannot be inferred, and residual confounding may persist despite adjustment for available clinical variables. The external validity of our results may also be limited by cohort-specific factors, including population characteristics and the distribution of ABO blood groups; therefore, replication in geographically and ethnically diverse SCLC populations is warranted. Second, inflammatory indices were derived from a single baseline complete blood count measurement, which may not fully capture dynamic immune–hematologic changes during treatment. Longitudinal assessment of inflammatory markers could provide more precise prognostic insights. Third, interaction analyses are inherently sensitive to sample size and model specification. In our cohort, some ABO subgroups—particularly blood group AB—were represented by a relatively small number of patients. Therefore, although the direction of the association was consistent across analyses, the effect estimates for the AB × high SIRI subgroup should be interpreted with caution, as small subgroup size may have contributed to wider confidence intervals and less stable point estimates. Accordingly, these findings require validation in larger, independent cohorts before firm subgroup-specific clinical inferences can be made. Finally, while mechanistic hypotheses (e.g., ABO–VWF–thrombosis biology or glycosylation-driven inflammation) are plausible and supported by indirect evidence, the causal chain in SCLC remains to be demonstrated in studies incorporating cytokines, coagulation markers, and longitudinal immune profiling [44]. Validation through large, prospective randomized controlled trials accounting for these limitations is necessary to clarify whether the ABO–inflammation interaction provides incremental prognostic value beyond established clinical variables and contributes to clinical decision-making.

## 5. Conclusions

This study demonstrates that systemic inflammatory indices are elevated in patients with small cell lung cancer who exhibit treatment non-response and poorer survival outcomes and that the prognostic impact of inflammation varies according to ABO blood group. Patients with blood group O displayed consistently lower inflammatory indices—particularly SIRI—compared with non-O groups. Our findings suggest that ABO-related host biology may modulate the clinical significance of systemic inflammation in SCLC. Incorporating ABO blood group status alongside inflammation-based biomarkers may provide a more biologically informed framework for risk assessment and prognostic evaluation.

## Figures and Tables

**Table 1:** Comparison of demographic and laboratory findings between ABO blood group in patients with SCLC.

Variables	ABO Blood Group		*p*
	0	Non-0	
	*n* = 49	*n* = 109	
Age, years	60.7 ± 10.2	62.4 ± 8.9	0.272
Male gender, *n* (%)	30 (61.2)	86 (78.9)	0.020 *
Weight, kg	73.6 ± 15.1	74.1 ± 12.3	0.830
BMI, kg/m^2^	26.9 ± 5.4	26.1 ± 4.4	0.295
Smoking, *n* (%)			
None	9 (18.4)	12 (11.0)	0.448
Smoker	24 (49.0)	56 (51.4)	
Ex-smoker	16 (32.7)	41 (37.6)	
CCI	6.0 (4.0–8.0)	8.0 (7.0–9.0)	0.001 *
Rh (+), *n* (%)	38 (77.6)	102 (93.6)	0.003 *
Laboratory findings			
Hemoglobin, g/dL	13.2 ± 1.7	12.8 ± 1.7	0.137
Leukocytes, ×10^9^/L	8.3 (6.5–10.6)	9.2 (7.4–10.9)	0.114
Neutrophils, ×10^9^/L	5.2 (3.7–6.9)	6.2 (5.0–8.0)	0.034 *
Lymphocytes, ×10^9^/L	2.0 (1.6–2.3)	1.7 (1.3–2.3)	0.096
Monocytes, ×10^9^/L	0.6 (0.5–0.8)	0.7 (0.6–0.9)	0.012 *
Platelets, ×10^9^/L	296.0 (235.0–350.0)	293.0 (243.0–392.0)	0.590
PLR	148.6 (116.2–185.5)	162.9 (117.5–243.5)	0.150
NLR	2.5 (1.8–3.7)	3.4 (2.3–5.0)	0.010 *
SII	792.0 (494.3–1094.0)	1133.0 (618.0–1636.0)	0.008 *
SIRI	1.7 (1.1–2.7)	2.4 (1.7–4.2)	0.003 *
CRP, mg/L	6.4 (2.3–13.0)	13.0 (6.4–46.0)	0.001 *

Data are mean ± standard deviation or median (IQR), or number (%). * A *p* value < 0.05 was considered statistically significant. Abbreviations: BMI, body mass index; CCI, Charlson Comorbidity Index; SII, systemic immune–inflammation index; SIRI, systemic inflammation response index; NLR, neutrophil-to-lymphocyte ratio; PLR, platelet-to-lymphocyte ratio; CRP, C-reactive protein.

**Table 2:** Comparison of clinical and outcome findings between ABO blood group in patients with SCLC.

Variables	ABO Blood Group		*p*
	0	Non-0	
	*n* = 49	*n* = 109	
Tumor location, *n* (%)			
Central	12 (24.5)	45 (41.3)	0.042 *
Peripheral	37 (75.5)	64 (58.7)	
Ki-67 PI, %	83.2 ± 13.0	84.1 ± 12.6	0.743
TNM stage at diagnosis, *n* (%)			
II	6 (12.2)	3 (2.8)	0.001 *
III	24 (49.0)	32 (29.4)	
IV	19 (38.8)	74 (67.9)	
Chemoradiotherapy, *n* (%)	27 (55.1)	27 (24.8)	<0.001 *
Metastasis area, *n* (%)			
Liver	7 (14.3)	38 (34.9)	0.008 *
Bone	16 (32.7)	51 (46.8)	0.096
Adrenal	3 (6.1)	18 (16.5)	0.075
Brain	7 (14.3)	25 (22.9)	0.211
Pancreas	1 (2.0)	3 (2.8)	0.792
ECOG, *n* (%)			
0	45 (91.8)	69 (63.3)	0.001 *
1	4 (8.2)	35 (32.1)	
2	0	5 (4.6)	
Chemotherapy, *n* (%)			
Cisplatin-based doublet	31 (63.3)	65 (59.6)	0.665
Carboplatin-based doublet	18 (36.7)	44 (40.4)	
Toxicity, *n* (%)	32 (65.3)	73 (67.0)	0.837
Grade 3–4	9 (18.4)	37 (33.9)	0.046 *
Response to chemotherapy			
Responders	45 (91.8)	71 (65.1)	<0.001 *
Non-responders	4 (8.2)	38 (34.9)	
Disease progression, *n* (%)	13 (26.5)	59 (54.1)	<0.001 *
PFS, months	55.0 (52.0–60.0)	42 (35–48)	<0.001 *
Mortality, *n* (%)	14 (28.6)	71 (65.1)	<0.001 *
Overall survival, months	56.0 (52.0–60.0)	46 (33–58)	<0.001 *

Data are presented as mean ± standard deviation, median (IQR), or number (%). * A *p* value < 0.05 was considered statistically significant. Abbreviations: ECOG, Eastern Cooperative Oncology Group performance status; PFS, progression-free survival; OS, overall survival.

**Table 3:** Diagnostic performance of inflammatory indices for predicting outcomes.

ROC Curve Findings	SII	SIRI	NLR	PLR
Non-response to CTx				
AUC ± SE	0.69 ± 0.05	0.73 ± 0.05	0.70 ± 0.05	0.57 ± 0.05
95% CI	0.59–0.79	0.63–0.83	0.60–0.80	0.47–0.68
Sensitivity	78.6%	72.0%	67.1%	36.2%
Specificity	61.2%	81.9%	78.6%	78.5%
Cut-off	>998.8	>2.1	≥3.8	>193.3
*p*-value	<0.001 *	<0.001 *	<0.001 *	0.177
Disease progression				
AUC ± SE	0.67 ± 0.04	0.74 ± 0.04	0.70 ± 0.04	0.60 ± 0.05
95% CI	0.59–0.75	0.66–0.82	0.61–0.78	0.51–0.69
Sensitivity	77.8%	79.2%	70.8%	51.4%
Specificity	53.5%	70.3%	66.3%	73.3%
Cut-off	>792.0	≥2.3	>3.1	≥181.7
*p*-value	<0.001 *	<0.001 *	<0.001 *	0.035 *
Mortality				
AUC ± SE	0.75 ± 0.04	0.80 ± 0.04	0.74 ± 0.04	0.65 ± 0.04
95% CI	0.67–0.83	0.73–0.87	0.66–0.82	0.56–0.74
Sensitivity	70.6%	77.1%	69.4%	68.2%
Specificity	75.3%	84.9%	75.3%	61.6%
Cut-off	≥998.8	≥2.6	≥3.4	>149.5
*p*-value	<0.001 *	<0.001 *	<0.001 *	0.001 *

Data are presented as area under the curve (AUC) ± standard error (SE), 95% confidence interval (CI), sensitivity (%), specificity (%), and optimal cut-off values. Optimal cut-off points were determined using the Youden index. * A *p* value < 0.05 was considered statistically significant. Abbreviations: SII, systemic immune–inflammation index; SIRI, systemic inflammation response index; NLR, neutrophil-to-lymphocyte ratio; PLR, platelet-to-lymphocyte ratio; AUC, area under the curve; SE, standard error; CI, confidence interval.

**Table 4:** Independent factors associated with non-response to chemotherapy.

Variables	Crude Regression	*p*	Multivariable Regression	*p*
	OR (95% CI)		OR (95% CI)	
Male gender, *n* (%)	2.70 (1.04–6.98)	0.040 *	–	–
ABO blood group, *n* (%)				
0	ref		ref	
A	5.11 (1.62–16.17)	0.005 *	3.68 (1.07–12.64)	0.019 *
B	7.87 (2.30–26.94)	0.001 *	8.72 (2.30–32.93)	0.002 *
AB	6.43 (1.30–31.79)	0.023 *	4.94 (1.03–29.31)	0.028 *
SIRI	1.62 (1.32–1.98)	<0.001 *	1.44 (1.09–1.90)	0.010 *
CRP	1.02 (1.01–1.03)	<0.001 *	1.03 (1.01–1.04)	0.011 *
ABO blood group × SIRI				
0 × SIRI	ref		ref	
A × SIRI	1.55 (1.02–2.47)	0.013 *	1.45 (1.04–2.26)	0.016 *
B × SIRI	1.93 (1.23–3.03)	0.005 *	1.85 (1.19–2.88)	0.008 *
AB × SIRI	1.79 (1.21–2.65)	0.004 *	1.71 (1.17–2.51)	0.011 *
TNM stage at diagnosis, *n* (%)				
II–III	ref		ref	
IV	5.26 (2.05–13.53)	<0.001 *	4.43 (1.39–14.05)	0.009 *
Chemoradiotherapy	0.29 (0.12–0.72)	<0.001 *	–	–
ECOG, *n* (%)				
0	ref		ref	
1–2	30.16 (11.45–79.43)	<0.001 *	23.44 (8.64–63.58)	<0.001 *

Age, BMI, smoking, CCI, tumor location, and chemotherapy type were adjusted in multivariable analyses. All variables significant in the crude regression analysis were entered into the multivariable model; variables that did not retain statistical significance are indicated by “–“. * A *p* value < 0.05 was considered statistically significant. Abbreviations: OR, odds ratio; CI, confidence interval; SIRI, systemic inflammation response index; CRP, C-reactive protein; ECOG, Eastern Cooperative Oncology Group performance status.

**Table 5:** Independent factors associated with disease progression in patients with SCLC.

Variables	Crude Regression		Multivariable Regression	
	HR (95% CI)	*p*	HR (95% CI)	*p*
CCI	1.41 (1.25–1.58)	<0.001 *	1.38 (1.18–1.60)	<0.001 *
ABO blood group				
0	ref		ref	
A	2.68 (1.41–5.10)	0.003 *	2.55 (1.31–4.68)	0.005 *
B	2.55 (1.22–5.25)	0.008 *	2.48 (1.15–4.29)	0.015 *
AB	6.45 (2.51–16.58)	<0.001 *	6.91 (2.70–17.68)	0.003 *
Rh (+)	3.51 (1.10–11.17)	0.033 *	–	-
SIRI	1.27 (1.15–1.39)	<0.001 *	1.24 (1.11–1.39)	<0.001 *
CRP	1.05 (1.01–1.10)	<0.001 *	1.06 (1.02–1.11)	0.005 *
ABO blood group × SIRI				
0 × SIRI	ref		ref	
A × SIRI	1.38 (1.12–1.71)	0.002 *	1.33 (1.09–1.62)	0.005 *
B × SIRI	1.24 (1.08–1.58)	0.011 *	1.22 (1.07–1.53)	0.017 *
AB × SIRI	1.46 (1.02–2.17)	0.018 *	1.55 (1.07–2.24)	0.020 *
Tumor location				
Central	ref		ref	
Peripheral	0.58 (0.36–0.93)	0.002 *	0.48 (0.31–0.76)	0.008 *
TNM stage at diagnosis				
II–III	ref		ref	
IV	4.17 (2.42–7.18)	<0.001 *	3.61 (2.08–6.26)	<0.001 *
Chemoradiotherapy, *n* (%)	0.25 (0.14–0.46)	<0.001 *	–	-
Response to CTx				
Responders	ref		ref	
Non-responders	3.64 (2.27–5.85)	<0.001 *	2.04 (1.17–3.55)	0.012 *
ECOG				
0	ref		ref	
1–2	2.90 (1.77–4.74)	<0.001 *	2.45 (1.56–3.84)	0.001 *
			−2Log Likelihood = 684.5, *p* < 0.001	

Age, gender, BMI, smoking, and chemotherapy type were adjusted in multivariable analyses. All variables significant in the crude regression analysis were entered into the multivariable model; variables that did not retain statistical significance are indicated by “–“. * A *p* value < 0.05 was considered statistically significant. Abbreviations: HR, hazard ratio; CI, confidence interval; CCI, Charlson Comorbidity Index; CRP, C-reactive protein; CTx, chemotherapy; ECOG, Eastern Cooperative Oncology Group performance status; SIRI, systemic inflammation response index.

**Table 6:** Independent factors associated with mortality.

Variables	Crude Regression		Multivariable Regression	
	HR (95% CI)	*p*	HR (95% CI)	*p*
Male gender	2.38 (1.34–4.23)	0.003 *	1.86 (1.03–3.35)	0.039 *
BMI	0.95 (0.90–1.00)	0.036 *	–	–
CCI	1.39 (1.24–1.55)	<0.001 *	1.35 (1.13–1.61)	0.001 *
ABO blood group				
0	ref		ref	
A	3.19 (1.73–5.86)	<0.001 *	1.98 (1.04–3.78)	0.022 *
B	2.64 (1.36–5.14)	0.004 *	2.04 (1.04–4.01)	0.024 *
AB	7.16 (2.80–18.32)	<0.001 *	3.48 (1.29–9.72)	0.014 *
Rh (+)	2.48 (1.00–6.12)	0.050 *	–	–
SIRI	1.22 (1.12–1.33)	<0.001 *	1.14 (1.03–1.25)	0.011 *
CRP	1.01 (1.00–1.01)	0.002 *		
ABO blood group × SIRI				
0 × SIRI	ref		ref	
A × SIRI	1.38 (1.12–1.71)	0.012 *	1.23 (1.02–1.48)	0.027 *
B × SIRI	1.24 (1.08–1.47)	0.021 *	1.12 (1.02–1.38)	0.032 *
AB × SIRI	1.55 (1.03–2.13)	0.014 *	1.47 (1.08–2.00)	0.016 *
Tumor location				
Central	ref		ref	
Peripheral	0.61 (0.40–0.94)	0.024 *	0.54 (0.33–0.91)	0.020 *
TNM stage at diagnosis				
II–II	ref		ref	
IV	4.06 (2.46–6.71)	<0.001 *	3.51 (2.11–5.84)	<0.001 *
Chemoradiotherapy, *n* (%)	0.29 (0.17–0.49)	<0.001 *	–	–
ECOG				
0	ref		ref	
1–2	3.13 (2.01–4.87)	<0.001 *	2.48 (1.57–3.92)	0.001 *
Response to CTx at 6 months				
Responders	ref		ref	
Non-responders	3.48 (2.26–5.35)	<0.001 *	2.30 (1.47–3.61)	0.001 *
Disease progression, *n* (%)	3.94 (2.44–6.37)	<0.001 *	1.81 (1.08–3.04)	0.024 *
			−2Log Likelihood = 688.2, *p* < 0.001	

Age, smoking, and chemotherapy type were adjusted in multivariable analyses. All variables significant in the crude regression analysis were entered into the multivariable model; variables that did not retain statistical significance are indicated by “–“. * A *p* value < 0.05 was considered statistically significant. Abbreviations: HR, hazard ratio; CI, confidence interval; CCI, Charlson Comorbidity Index; CRP, C-reactive protein; CTx, chemotherapy; ECOG, Eastern Cooperative Oncology Group performance status; SIRI, systemic inflammation response index.

## Data Availability

The data that support the findings of this study are available on request from the corresponding author due to privacy and ethical reasons.

## Supplementary Materials

The following supporting information can be downloaded at https://www.mdpi.com/article/10.3390/biomedicines14051051/s1, Table S1: Demographic and clinical characteristics of SCLC patients; Table S2: Comparison of demographic and clinical characteristics according to response to chemotherapy at 6 months in SCLC patients; Table S3: Demographic and clinical parameters associated with disease progression; Table S4: Demographic and clinical parameters associated with mortality.
