# Supplementary material for: Prognostic Impact of ABO/Rh Blood Group–Systemic Inflammation Indices Interactions in Small-Cell Lung Cancer
Source: Biomedicines. 2026 May 6;14(5):1051. doi: 10.3390/biomedicines14051051 (PMC13204975; doi:10.3390/biomedicines14051051)
Supplement: Supplementary file 1 [file biomedicines-14-01051-s001.zip › biomedicines-4196228-supplementary.pdf]

Supplementary Table S1. Demographic and clinical characteristics of SCLC patients.

Variables	All population n = 158
Age, years	61.9 ± 9.3
Male gender, n (%)	116 (73.4)
Weight, kg	73.9 ± 13.2
BMI, kg/m^2	26.3 ± 4.8
Smoking, n (%)	
None	21 (13.3)
Smoker	80 (50.6)
Ex-smoker	57 (36.1)
Comorbidities, n (%)	90 (57.0)
Hypertension	65 (41.1)
Diabetesmellitus	33 (20.9)
CHF	8 (5.1)
CAD	29 (18.4)
COPD	8 (5.1)
CRF	1 (0.6)
CCI	8.0 (6.09.0)
ABO blood group, n (%)	
0	49 (31.0)
A	64 (40.5)
B	34 (21.5)
AB	11 (7.0)
Rh (+), n (%)	140 (88.6)
Tumor location, n (%)	
Central	57 (36.1)
Peripheral	101 (63.9)
Ki-67 PI, %	83.8 ± 12.7
TNM stage at diagnosis, n (%)	
II	9 (5.7)
III	56 (35.4)
IV	93 (58.9)
Treatment before metastasis, n (%)	
Chemotherapy	–
Chemoradiotherapy	54 (34.2)
Metastasis area, n (%)	
Liver	45 (28.5)
Bone	67 (42.4)
Adrenal	21 (13.3)
Brain	32 (20.3)
Pancreas	4 (2.5)
ECOG, n (%)	
0	114 (72.2)
1-2	39 (24.7)
CTx, n (%)	
Cisplatin-based doublet	96 (60.8)
Carboplatin-based doublet	62 (39.2)
Response to CTx at 6 months	
Responders	116 (73.4)
Non-responders	42 (26.6)
Toxicity, n (%)	105 (66.5)
Grade 3-4	46 (29.1)
Disease progression, n (%)	72 (45.6)
PFS, months	54.0 (47.0 – 60.0)
Mortality, n (%)	85 (53.8)
Overall survival, months	53.0 (45.0 – 60.0)
Follow-up time, months	60.0 (46.0 – 60.0)

Data are mean ± standard deviation or median (IQR), or number (%). Abbreviations: BMI, body mass index; CHF, congestive heart failure; CAD, coronary artery disease; COPD, chronic obstructive pulmonary disease; CRF, chronic renal failure; CCI, Charlson Comorbidity Index; TNM, Tumor–Node–Metastasis classification; ECOG, Eastern Cooperative Oncology Group performance status; CTx, chemotherapy; PFS, progression-free survival.

Supplementary Table S2. Comparison of demographic and clinical characteristics according to response to chemotherapy at 6 months in SCLC patients.

Variables	Response to CTx		p
	Responders n = 116	Non-responders n = 42	
Age, years	62.3 ± 9.1	60.8 ± 10.0	0.392
Male gender, n (%)	80 (69.0)	36 (85.7)	0.035*
Weight, kg	73.4 ± 13.0	75.3 ± 13.5	0.428
BMI, kg/m^2	26.5 ± 5.0	25.9 ± 4.1	0.469
Smoking, n (%)			
None	18 (15.5)	3 (7.1)	
Smoker	55 (47.4)	25 (59.5)	0.302
Ex-smoker	43 (37.1)	14 (33.3)	
CCI	8.0 (6.0 – 9.0)	8.0 (7.0 – 9.0)	0.268
ABO blood group, n (%)			
0	45 (38.8)	4 (9.5)	
A	44 (37.9)	20 (47.6)	0.002*
B	20 (17.2)	14 (33.3)	
AB	7 (6.0)	4 (9.5)	
Rh (+), n (%)	100 (86.2)	40 (95.2)	0.195
Laboratory findings			
Hemoglobin, g/dL	12.8 ± 1.7	13.3 ± 1.9	0.161
Leukocytes, $\times 10^9/L$	8.6 (6.7 – 10.4)	10.4 (8.7 – 12.0)	<0.001*
Neutrophils, $\times 10^9/L$	5.5 (4.2 – 7.2)	7.7 (6.3 – 8.7)	<0.001*
Lymphocytes, $\times 10^9/L$	1.9 (1.5 – 2.4)	1.7 (1.2 – 2.3)	0.179
Monocytes, $\times 10^9/L$	0.7 (0.6 – 0.8)	0.8 (0.6 – 1.1)	0.016*
Platelets, $\times 10^9/L$	286.0 (240.5 – 383.5)	308.0 (230.0 – 398.0)	0.685
SII	879.8 (553.8 – 1257.3)	1372.1 (1036.4 – 2135.3)	<0.001*
SIRI	1.9 (1.3 – 2.5)	4.2 (2.1 – 5.4)	<0.001*
NLR	2.8 (2.1 – 4.0)	4.5 (3.4 – 6.7)	<0.001*
PLR	150.8 (113.0 – 218.8)	173.2 (123.9 – 282.9)	0.159
CRP, mg/L	8.0 (3.0 – 20.0)	32.5 (9.0 – 75.8)	<0.001*
Tumor location, n (%)			
Central	39 (33.6)	18 (42.9)	0.286
Peripheral	77 (66.4)	24 (57.1)	
Ki-67 PI, %	84.9 ± 11.1	81.2 ± 15.7	0.218
TNM stage at diagnosis, n (%)			
II	9 (7.8)	0 (0.0)	
III	50 (43.1)	6 (14.3)	<0.001*
IV	57 (49.1)	36 (85.7)	
Chemoradiotherapy, n (%)	47 (40.5)	7 (16.7)	0.005*
Metastasis area, n (%)			
Liver	24 (20.7)	21 (50.0)	<0.001*
Bone	44 (37.9)	23 (54.8)	0.059
Adrenal	12 (10.3)	9 (21.4)	0.070
Brain	23 (19.8)	9 (21.4)	0.825
Pancreas	3 (2.6)	1 (2.4)	0.942
ECOG, n (%)			
0	104 (89.7)	10 (23.8)	<0.001*
1-2	10 (8.6)	29 (69.0)	
CTx, n (%)			
Cisplatin-based doublet	66 (56.9)	30 (71.4)	0.098
Carboplatin-based doublet	50 (43.1)	12 (28.6)	
Toxicity, n (%)	82 (70.7)	23 (54.8)	0.061
Grade 3-4	36 (31.0)	10 (23.8)	0.377
Disease progression, n (%)	41 (35.3)	31 (73.8)	<0.001*
PFS, months	55.0 (52.0 – 60.0)	21.0 (16.0 – 26.0)	<0.001*
Mortality, n (%)	47 (40.5)	38 (90.5)	<0.001*
Overall survival, months	58.0 (56.0 – 60.0)	28.0 (19.0 – 36.0)	0.002*

Data are mean ± standard deviation or median (IQR), or number (%). Abbreviations: see Supplementary Table 1. SII, systemic immune-inflammation index; SIRI, systemic inflammation response index; NLR, neutrophil-to-lymphocyte ratio; PLR, platelet-to-lymphocyte ratio; CRP, C-reactive protein.

Supplement Table S3. Demographic and clinical parameters associated with disease progression.

Variables	Disease progression		Crude regression	
	No<br>n = 86	Yes<br>n = 72	HR (95% CI)	p
Age, years	62.7 ± 8.4	60.9 ± 10.3	0.99 (0.97 – 1.02)	0.668
Male gender, n (%)	60 (69.8)	56 (77.8)	1.65 (0.95 – 2.88)	0.077
Weight, kg	73.3 ± 12.9	74.7 ± 13.5	1.00 (0.98 – 1.02)	0.933
BMI, kg/m2	26.6 ± 5.1	26.0 ± 4.3	0.95 (0.90 – 1.00)	0.055
Smoking, n (%)				
None	15 (17.4)	6 (8.3)	ref	
Smoker	40 (46.5)	40 (55.6)	1.21 (51 – 2.89)	0.662
Ex-smoker	31 (36.0)	26 (36.1)	1.01 (0.40 – 2.38)	0.954
CCI	7.0 (4.0 – 8.0)	8.0 (7.0 – 9.0)	1.41 (1.25 – 1.58)	<0.001*
ABO blood group, n (%)				
0	36 (41.9)	13 (18.1)	ref	
A	31 (36.0)	33 (45.8)	2.68 (1.41 – 5.10)	0.003*
B	15 (17.4)	19 (26.4)	2.59 (1.28 – 5.27)	0.008*
AB	4 (4.7)	7 (9.7)	6.45 (2.51 – 16.58)	<0.001*
Rh (+), n (%)	71 (82.6)	69 (95.8)	3.51 (1.10 – 11.17)	0.033*
Laboratory findings				
Hemoglobin, g/dL	12.7 ± 1.6	13.2 ± 1.8	1.09 (0.95 – 1.26)	0.215
Leukocytes, ×10⁹/L	8.5 (6.6 – 10.0)	9.9 (8.1 – 12.2)	1.07 (1.02 – 1.12)	0.003*
Neutrophils, ×10⁹/L	5.4 (3.8 – 7.0)	6.9 (5.4 – 8.5)	1.20 (1.11 – 1.31)	<0.001*
Lymphocytes, ×10⁹/L	1.9 (1.5 – 2.5)	1.7 (1.2 – 2.3)	0.82 (0.60 – 1.14)	0.241
Monocytes, ×10⁹/L	0.6 (0.5 – 0.8)	0.8 (0.6 – 1.1)	3.99 (1.73 – 9.19)	0.001*
Platelets, ×10⁹/L	286.0 (238.8 – 392.0)	297.5 (239.5 – 383.5)	1.06 (0.99 – 1.03)	0.622
SII	726.8 (504.5 – 1243.1)	1181.7 (872.7 – 1753.1)	1.05 (1.03 – 1.07)	<0.001*
SIRI	1.7 (1.2 – 2.6)	3.5 (2.0 – 4.5)	1.27 (1.15 – 1.39)	<0.001*
NLR	2.5 (2.0 – 3.8)	3.8 (2.7 – 5.5)	1.16 (1.08 – 1.25)	<0.001*
PLR	148.7 (112.3 – 194.6)	182.0 (126.1 – 252.8)	1.03 (1.01 – 1.08)	0.012*
CRP, mg/L	8.0 (2.8 – 21.2)	14.0 (8.0 – 57.0)	1.01 (1.00 – 1.01)	<0.001*
Tumor location, n (%)				
Central	25 (29.1)	32 (44.4)	ref	
Peripheral	61 (70.9)	40 (55.6)	0.58 (0.36 – 0.93)	0.002*
Ki-67 PI, %	82.4 ± 10.5	85.3 ± 14.6	1.00 (0.98 – 1.03)	0.817
TNM stage at diagnosis, n (%)				
II-III	47 (54.7)	18 (25.0)	ref	
IV	39 (45.3)	54 (75.0)	4.05 (2.35 – 6.98)	<0.001*
Chemoradiotherapy, n (%)	40 (46.5)	14 (19.4)	0.25 (0.14 – 0.46)	<0.001*
Metastasis area, n (%)				
Liver	17 (19.8)	28 (38.9)	2.77 (1.71 – 4.49)	<0.001*
Bone	26 (30.2)	41 (56.9)	2.49 (1.56 – 3.99)	<0.001*
Adrenal	9 (10.5)	12 (16.7)	1.77 (0.95 – 3.28)	0.273
Brain	17 (19.8)	15 (20.8)	1.46 (0.82 – 2.60)	0.293
Pancreas	0 (0.0)	4 (5.6)	1.83 (0.67 – 5.04)	0.289
ECOG, n (%)				
0	72 (83.7)	42 (58.3)	ref	
1-2	12 (14.0)	27 (37.5)	2.90 (1.77 – 4.74)	<0.001*
CTx, n (%)				
Cisplatin-based doublet	57 (66.3)	39 (54.2)	ref	
Carboplatin-based doublet	29 (33.7)	33 (45.8)	1.50 (0.94 – 2.38)	0.088
Response to CTx at 6 months				
Responders	75 (87.2)	41 (56.9)	ref	
Non-responders	11 (12.8)	31 (43.1)	3.64 (2.27 – 5.85)	<0.001*
Toxicity, n (%)	52 (60.5)	53 (73.6)	1.26 (0.74 – 2.12)	0.392
Grade 3-4	21 (24.4)	25 (34.7)	1.40 (0.86 – 2.27)	0.179
Mortality, n (%)	23 (26.7)	62 (86.1)	–	–

Data are mean ± standard deviation or median (IQR), or number (%). Abbreviations: see Supplementary Table 1.HR, hazard ratio; CI, confidence invertal; SII, systemic immune-inflammation index; SIRI, systemic inflammation response index; NLR, neutrophil-to-lymphocyte ratio; PLR, platelet-to-lymphocyte ratio; CRP, C-reactive protein.

Supplement Table S4. Demographic and clinical parameters associated with mortality.

Variables	Alive n=73	Deceased n=85	Crude regression HR (95% CI)	p
Age, years	61.3 ± 9.3	62.4 ± 9.4	1.02 (0.99 – 1.04)	0.172
Male gender, n (%)	45 (61.6)	71 (83.5)	2.38 (1.34 – 4.23)	0.003*
Weight, kg	72.9 ± 12.8	74.8 ± 13.5	1.00 (0.98 – 1.02)	0.960
BMI, kg/m2	26.8 ± 4.8	26.0 ± 4.8	0.95 (0.90 – 1.00)	0.036*
Smoking, n (%)				
None	12 (16.4)	9 (10.6)	ref	
Smoker	35 (47.9)	45 (52.9)	1.19 (0.58 – 2.43)	0.626
Ex-smoker	26 (35.6)	31 (36.5)	1.05 (0.84 – 1.26)	0.896
CCI	7.0 (4.0 – 8.0)	8.0 (7.0 – 9.0)	1.39 (1.24 – 1.55)	<0.001*
ABO blood group, n (%)				
0	35 (47.9)	14 (16.5)	ref	
A	23 (31.5)	41 (48.2)	3.19 (1.73 – 5.86)	<0.001*
B	11 (15.1)	23 (27.1)	2.64 (1.36 – 5.14)	0.004*
AB	4 (5.5)	7 (8.2)	7.16 (2.80 – 18.32)	<0.001*
Rh (+), n (%)	60 (82.2)	80 (94.1)	2.48 (1.00 – 6.12)	0.050*
Laboratory findings				
Hemoglobin, g/dL	12.8 ± 1.6	13.0 ± 1.8	1.03 (0.91 – 1.18)	0.611
Leukocytes, $\times 10^9$/L	8.1 (6.5 – 9.9)	9.9 (8.1 – 12.2)	1.05 (1.01 – 1.10)	0.022*
Neutrophils, $\times 10^9$/L	5.0 (3.8 – 6.5)	7.1 (5.5 – 8.5)	1.17 (1.08 – 1.26)	<0.001*
Lymphocytes, $\times 10^9$/L	2.0 (1.6 – 2.6)	1.7 (1.2 – 2.3)	0.81 (0.60 – 1.10)	0.174
Monocytes, $\times 10^9$/L	0.6 (0.5 – 0.8)	0.8 (0.6 – 1.1)	3.99 (1.85 – 8.60)	<0.001*
Platelets, $\times 10^9$/L	282.0 (235.0 – 359.0)	304.0 (243.0 – 402.0)	1.01 (0.98 – 1.03)	0.371
SII	637.5 (497.7 – 979.0)	1243.1 (908.3 – 1946.9)	1.04 (1.02 – 1.06)	<0.001*
SIRI	1.8 (1.2 – 2.5)	3.5 (2.0 – 4.5)	1.22 (1.12 – 1.33)	<0.001*
NLR	2.3 (1.9 – 3.2)	4.0 (2.8 – 5.7)	1.11 (1.05 – 1.19)	<0.001*
PLR	138.1 (111.0 – 179.1)	182.3 (135.7 – 248.5)	1.04 (1.01 – 1.10)	0.008*
CRP, mg/L	6.6 (2.5 – 12.8)	20.0 (8.0 – 46.0)	1.01 (1.00 – 1.01)	0.002*
Tumor location, n (%)				
Central	20 (27.4)	37 (43.5)	ref	
Peripheral	53 (72.6)	48 (56.5)	0.61 (0.40 – 0.94)	0.024*
Ki-67 PI, %	82.8 ± 10.3	84.6 ± 14.3	1.00 (0.98 – 1.02)	0.973
TNM stage at diagnosis, n (%)				
II--II	44 (60.3)	21 (24.7)	ref	
IV	29 (39.7)	64 (75.3)	4.06 (2.46 – 6.71)	<0.001*
Chemoradiotherapy, n (%)	36 (49.3)	18 (21.2)	0.29 (0.17 – 0.49)	<0.001*
Metastasis area, n (%)				
Liver	12 (16.4)	33 (38.8)	2.58 (1.65 – 4.02)	<0.001*
Bone	21 (28.8)	46 (54.1)	2.09 (1.36 – 3.21)	<0.001*
Adrenal	5 (6.8)	16 (18.8)	2.06 (1.19 – 3.56)	0.009*
Brain	12 (16.4)	20 (23.5)	1.58 (0.95 – 2.61)	0.076
Pancreas	1 (1.4)	3 (3.5)	1.16 (0.37 – 3.67)	0.804
ECOG, n (%)				
0	69 (94.5)	45 (52.9)	ref	
1	3 (4.1)	36 (42.4)	3.13 (2.01 – 4.87)	<0.001*
2	1 (1.4)	4 (4.7)	5.36 (1.89 – 15.19)	0.002*
CTx, n (%)				
Cisplatin-based doublet	42 (57.5)	54 (63.5)	Ref	
Carboplatin-based doublet	31 (42.5)	31 (36.5)	1.02 (0.65 – 1.58)	0.935
Response to CTx at 6 months				
Responders	69 (94.5)	47 (55.3)	Ref	
Non-responders	4 (5.5)	38 (44.7)	3.48 (2.26 – 5.35)	<0.001*
Toxicities	51 (69.9)	54 (63.5)	0.77 (0.49 – 1.19)	0.236
Grade 3-4	17 (23.3)	29 (34.1)	1.38 (0.88 – 2.17)	0.158
Disease progression, n (%)	10 (13.7)	62 (72.9)	3.94 (2.44 – 6.37)	<0.001*

Data are mean ± standard deviation or median (IQR), or number (%). Abbreviations: see Supplementary Table 1. SII, systemic immune-inflammation index; SIRI, systemic inflammation response index; NLR, neutrophil-to-lymphocyte ratio; PLR, platelet-to-lymphocyte ratio; CRP, C-reactive protein.
